# Supplementary material for: Comprehensive Evaluation of the m6A Regulator Prognostic Risk Score in the Prediction of Immunotherapy Response in Clear Cell Renal Cell Carcinoma
Source: Front Immunol. 2022 Jun 17;13:818120. doi: 10.3389/fimmu.2022.818120 (PMC9248360; doi:10.3389/fimmu.2022.818120)

Supplementary Figure Legends

SUPPLEMENTARY FIGURE 1 | (A) GEO database verifies the difference of m^{6}A RNA methylation regulators expression between renal cancer and normal tissues (GSE40435 and GSE53757). **(B)** Representative IHC images of two m^{6}A RNA methylation regulators expression in renal cancer and normal tissue. **(C)** UMAP embedding of transcriptional profiles from all patients and samples (n = 65535). Each dot represents a single cell, and colors represent clusters denoted by inferred cell type. **(D)** Dot plot analysis of key markers to define the 7 identified epithelial cell subsets. (*p < 0.05; **p < 0.01; ***p < 0.001; ****p < 0.0001; ns, not significant)

SUPPLEMENTARY FIGURE 2 | (A) Partial likelihood deviance for tuning the parameter selection in the LASSO regression model in TCGA-ccRCC cohort. **(B)** The prognostic analyses for 23 m^{6}A RNA methylation regulators in the TCGA-ccRCC cohort using the univariate Cox regression model. **(C)** Prognostic value of MRPRS score in multiple cancer cohorts.

SUPPLEMENTARY FIGURE 3 | (A) The MRPRS score between response and non-response groups in IMvigor210 (BLCA). **(B)** The MRPRS score between response and non-response groups in PRJEB23709 (SKCM). **(C)** Kaplan-Meier analysis of patients between high and low MRPRS score groups in IMvigor210 (BLCA). **(D)** Kaplan-Meier analysis of patients between high and low MRPRS score groups in phs000452 (SKCM). **(E)** Kaplan-Meier analysis of patients between high and low MRPRS score groups in PRJEB23709 (SKCM).

SUPPLEMENTARY FIGURE 4 | (A) Heatmap of eight candidate MRPRS score-related genes in the high and low groups, annotated with corresponding clinicopathologic characteristics. **(B)** A volcano plot was drawn based on the DEGs between high and low MRPRS score groups. **(C)** The PPI network was constructed by PPI pairs based on the STRING database from top50 DEGs. **(D-E)** GO and KEGG analysis of DEGs between high and low MRPRS score groups.

SUPPLEMENTARY FIGURE 5 | (A-C) The fraction of tumor-infiltrating immune cells in high and low MRPRS score groups using the CIBERSORT, ssGSEA and Xcell algorithmMCP_counter, cibersort, ssGSEA and xcell algorithm. (*p < 0.05; **p < 0.01; ***p < 0.001; ****p < 0.0001)

SUPPLEMENTARY FIGURE 6 | (A) Mutation status of high MRPRS score group in the TCGA-ccRCC cohort. **(B)** Mutation status of low MRPRS score group in the TCGA-ccRCC cohort. **(C)** Somatic mutation genes interaction networks by high MRPRS score group in the TCGA-ccRCC cohort. **(D)** Somatic mutation genes interaction networks by low MRPRS score group in the TCGA-ccRCC cohort.

SUPPLEMENTARY FIGURE 7 | (A-B) Kaplan-Meier analysis (OS) of patients between high and low MRPRS score groups in TCGA-ccRCC and TCGA-pan-cancer cohorts. **(C)** Kaplan-Meier analysis (PFS) of patients between high and low MRPRS score groups in TCGA-ccRCC and TCGA-pan-cancer cohorts.

Supplementary Figure 1

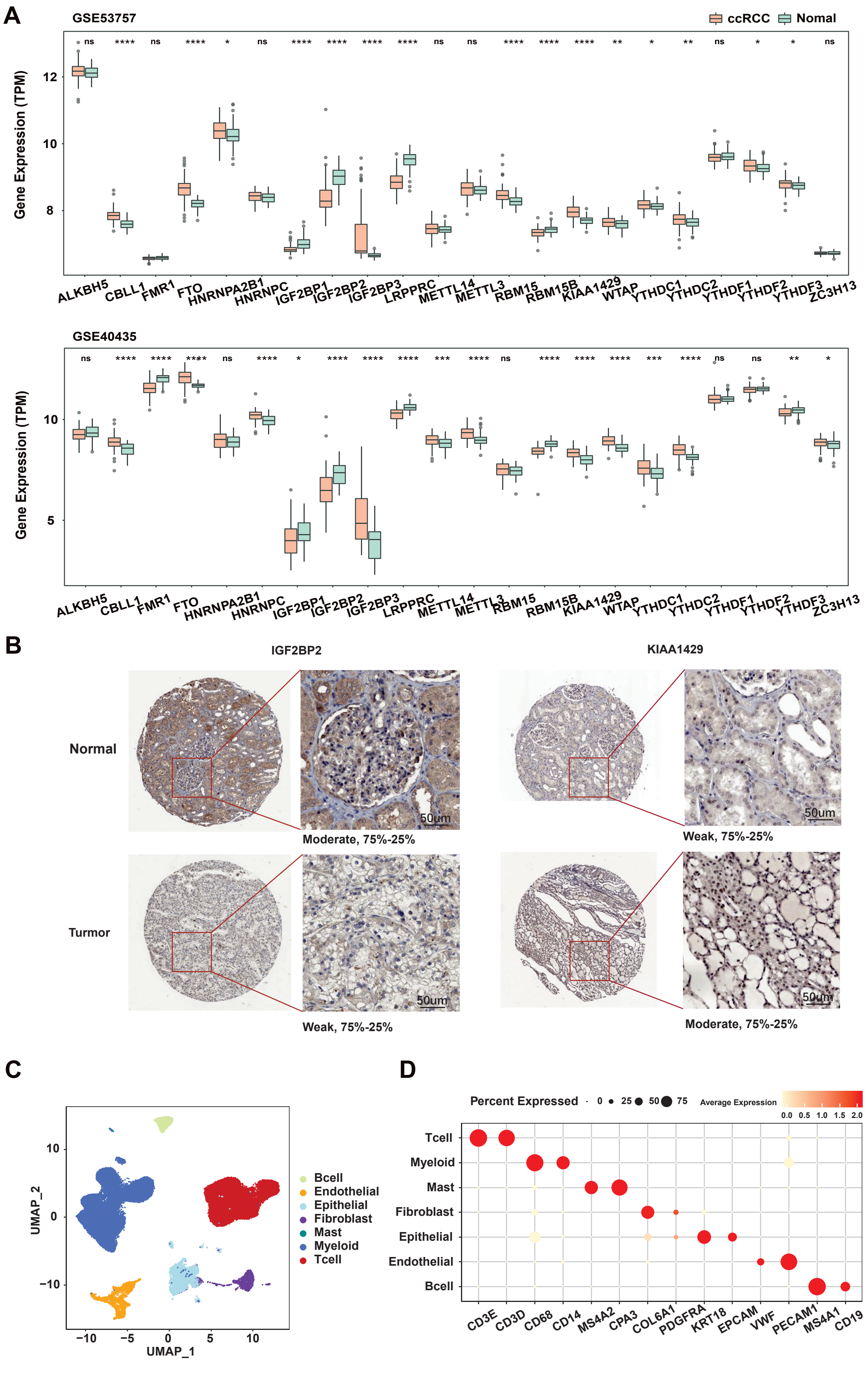

Supplementary Figure 2

A

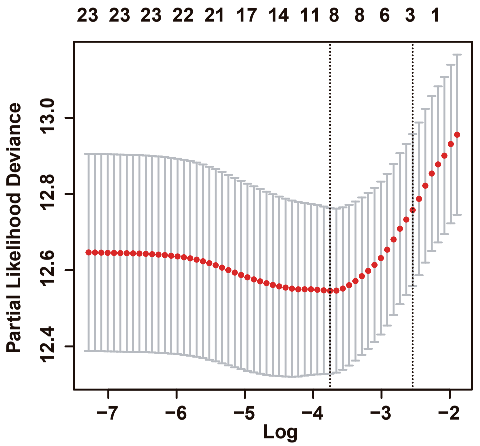

B

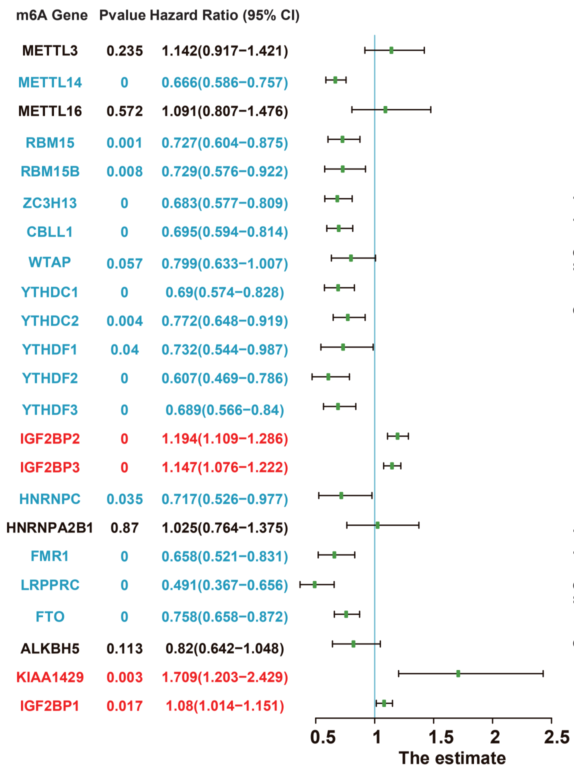

C

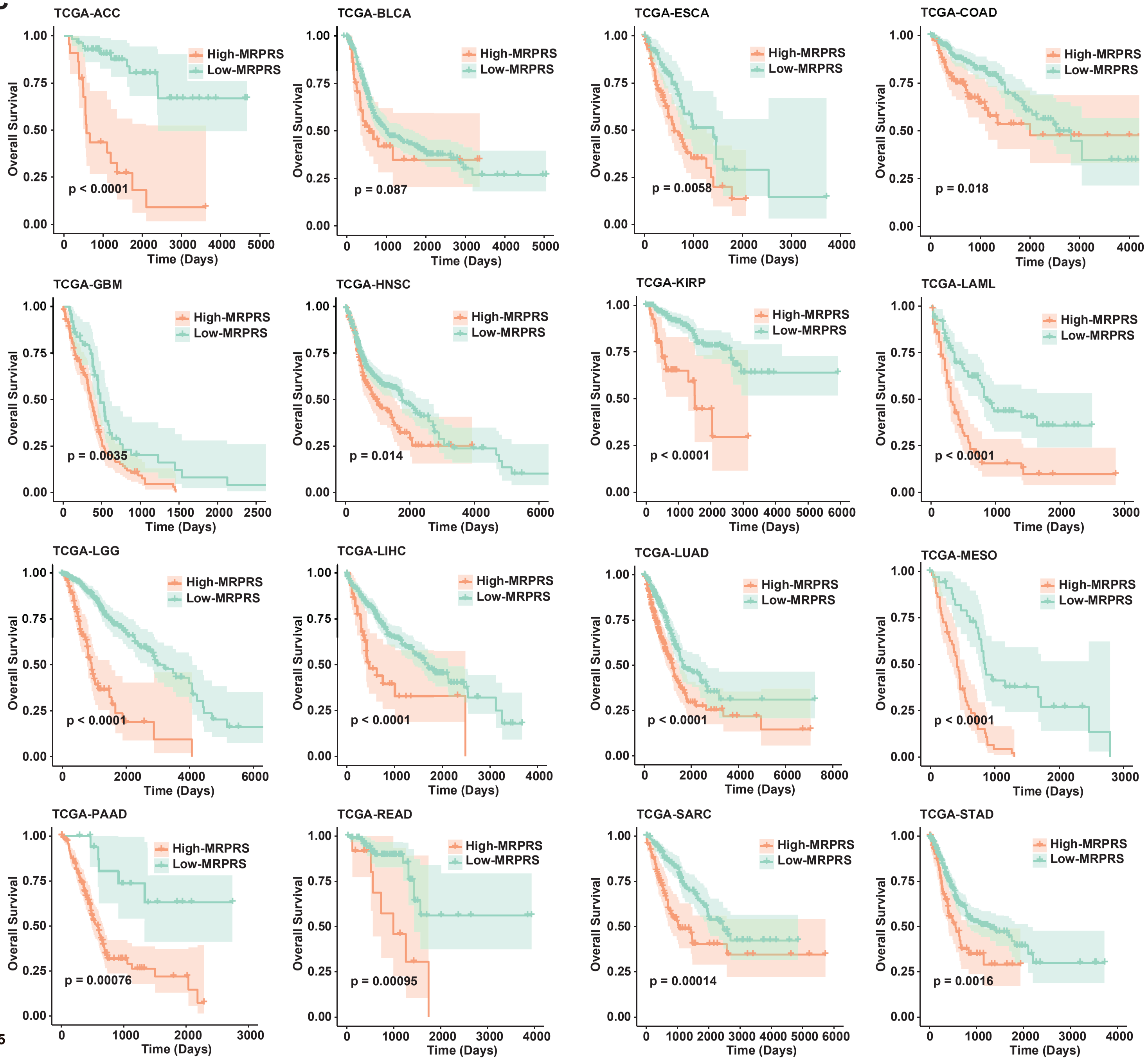

Supplementary Figure 3

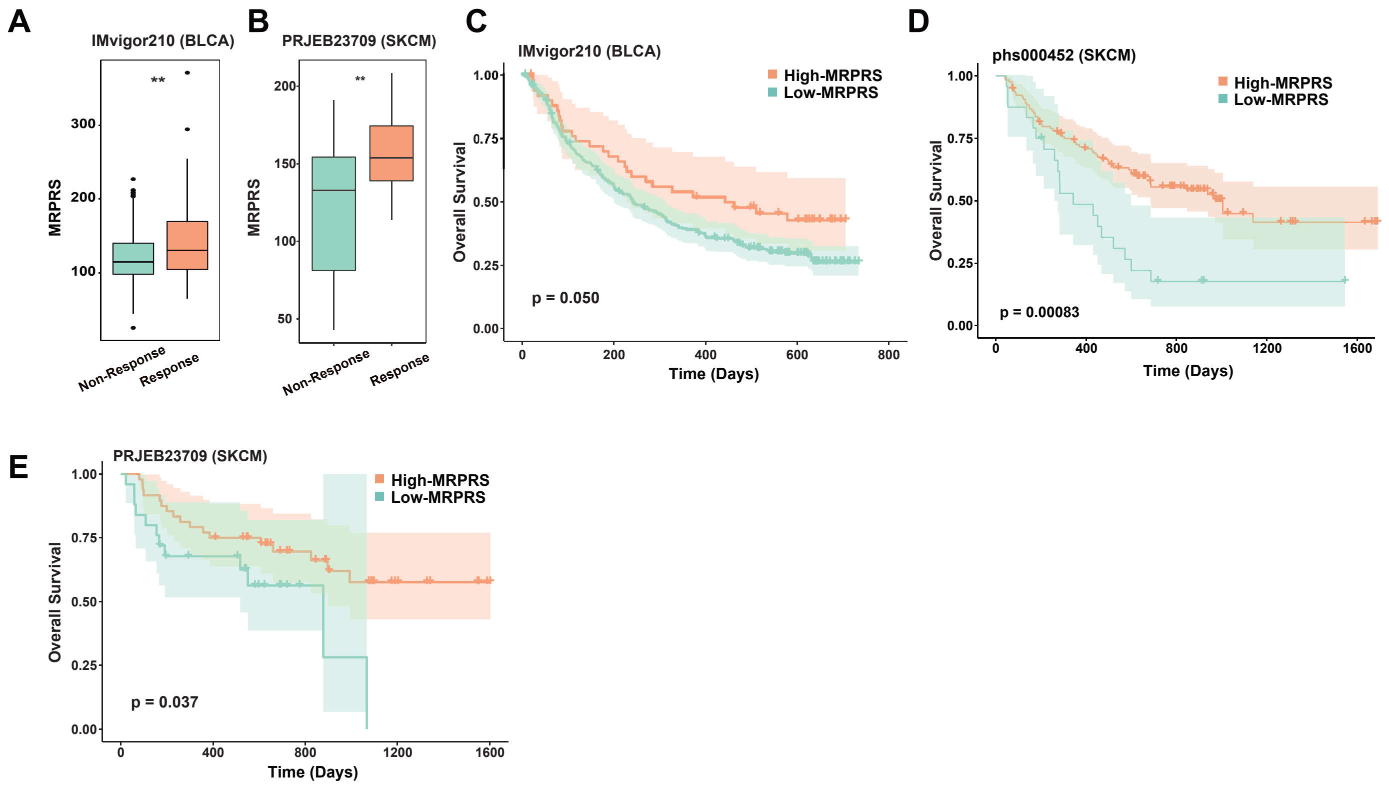

Supplementary Figure 4

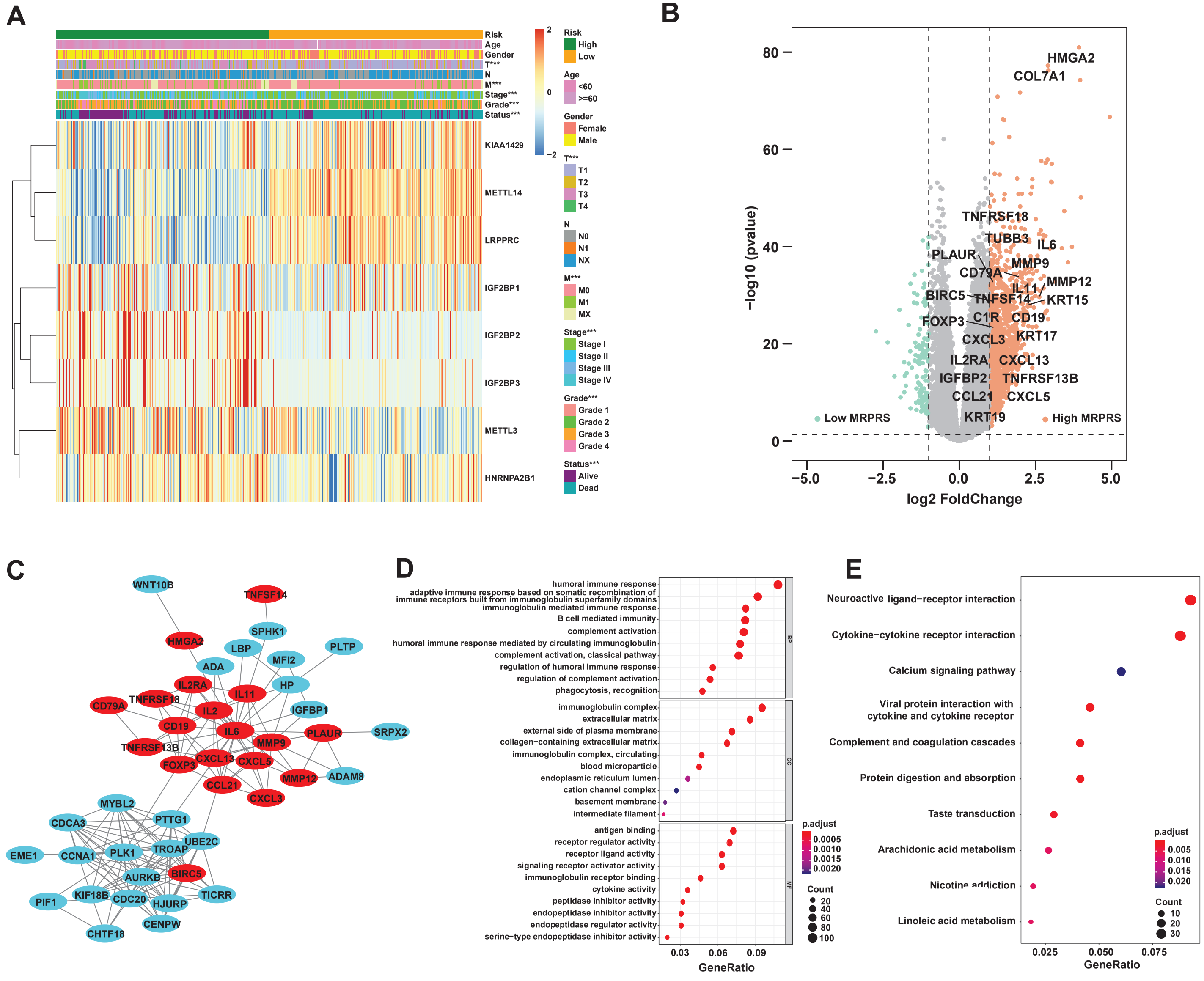

Supplementary Figure 5

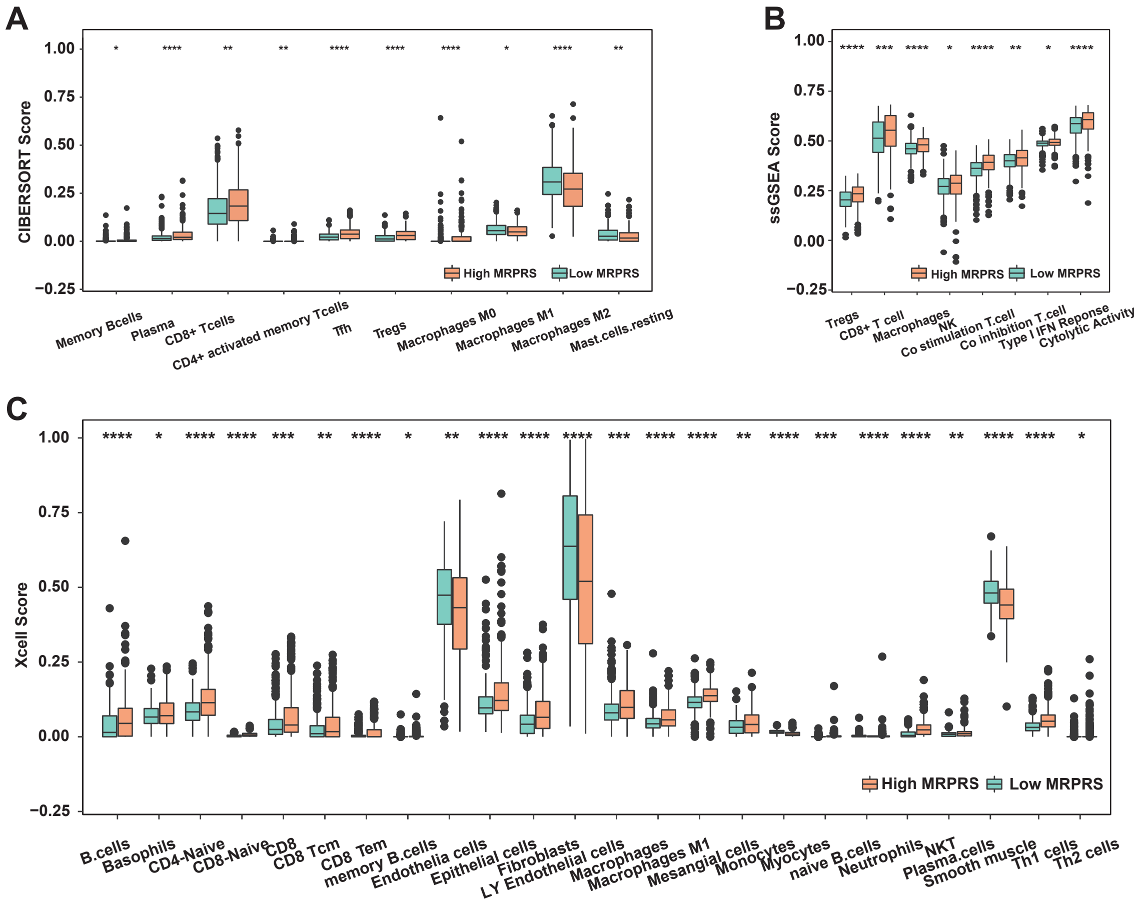

Supplementary Figure 6

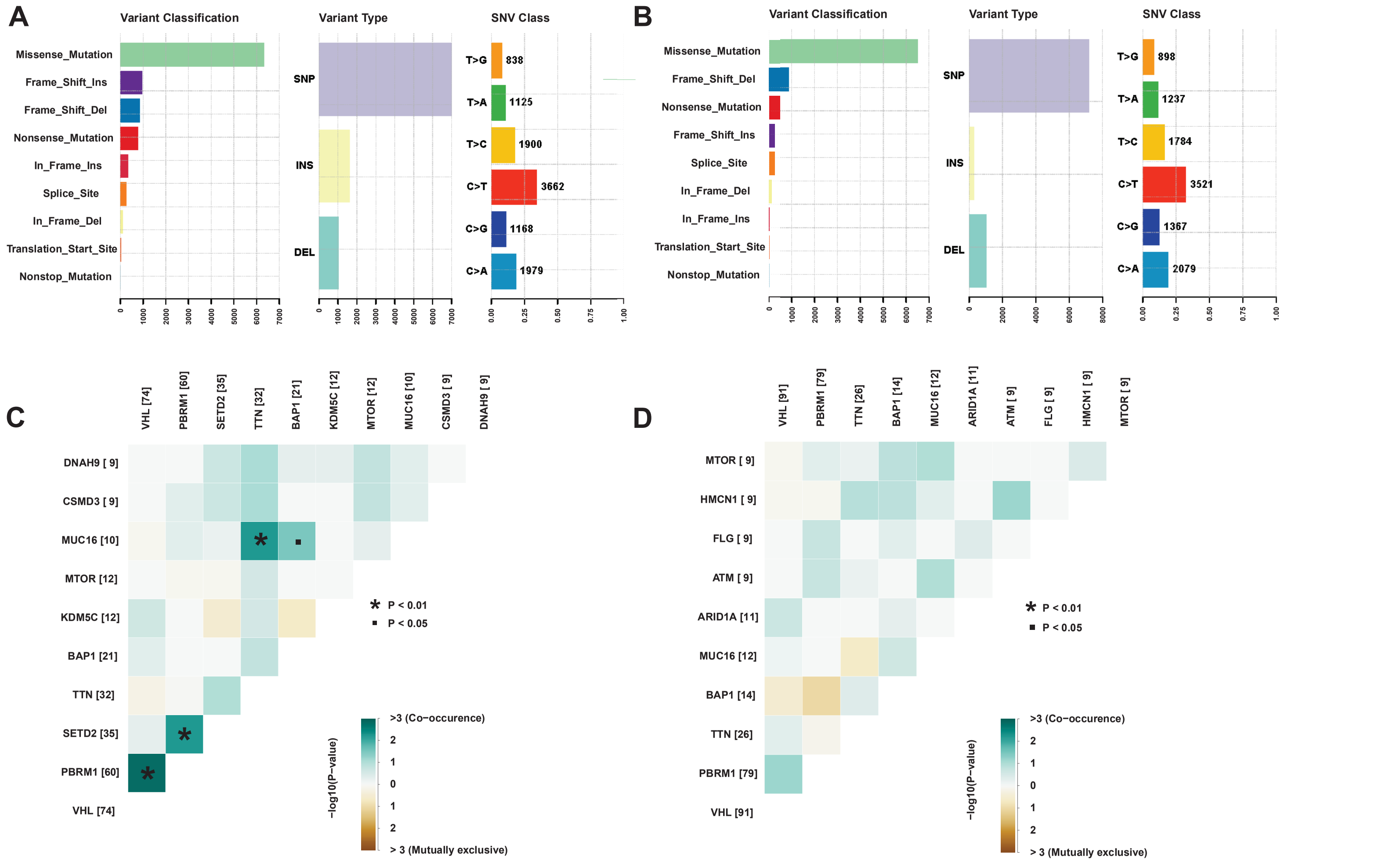

Supplementary Figure 7

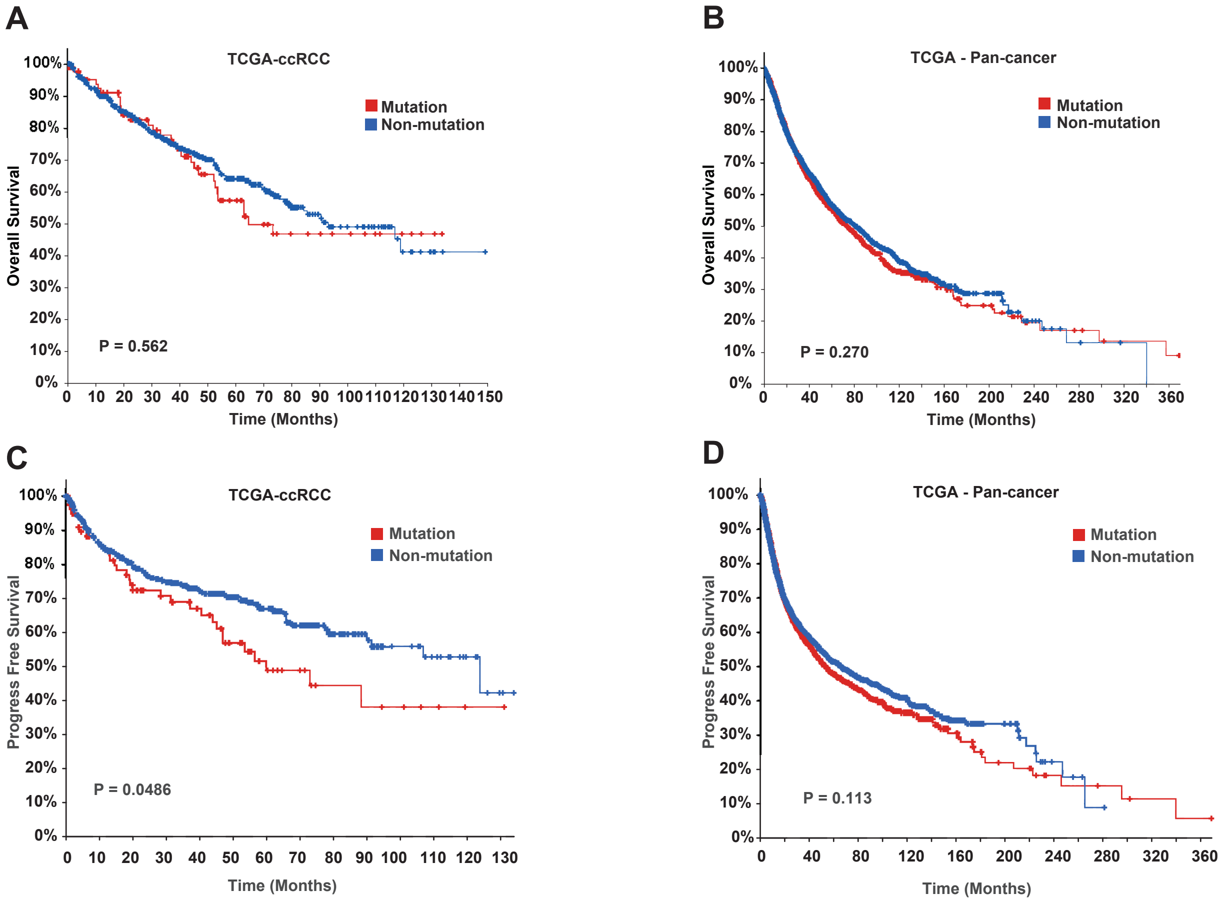

Supplement: Supplementary file 1 [file Image_1.pdf]
