# Supplementary material for: Comprehensive Evaluation of the m6A Regulator Prognostic Risk Score in the Prediction of Immunotherapy Response in Clear Cell Renal Cell Carcinoma
Source: Front Immunol. 2022 Jun 17;13:818120. doi: 10.3389/fimmu.2022.818120 (PMC9248360; doi:10.3389/fimmu.2022.818120)
Supplement: Supplementary file 2 [file Table_1.docx]

| PMID | Accession | Cancer Type | Patient number  (Case) |
| --- | --- | --- | --- |
| / | TCGA-ccRCC | ccRCC | 529 |
| [24962026](https://www.ncbi.nlm.nih.gov/pubmed/24962026) | GSE53757 | ccRCC | 72 |
| [23526956](https://www.ncbi.nlm.nih.gov/pubmed/23526956) | GSE40435 | ccRCC | 101 |
| 22626276 | GSE29609 | ccRCC | 39 |
| 25583177 | E-MTAB-3267 | metastatic ccRCC | 53 |
| 30753825 | PRJEB23709 | melanoma | 73 |
| 29132144 | \| phs000452 \| \| --- \| | metastatic melanoma | 164 |
| 32472114 | / | metastatic RCC | 53 |
| 32895571 | [NCT02684006](https://clinicaltrials.gov/ct2/show/NCT02684006) | metastatic RCC | 354 |
| 29301960 | / | metastatic ccRCC | 33 |
| 29337640 | / | NSCLC | 41 |
| 30643254 | / | advanced cancer | 1644 |
| 30150660 | // | solid tumors | 239 |
| 29443960 | IMvigor210 | metastatic urothelial cancer | 348 |

Abbreviations: Renal cell carcinoma, RCC; clear cell RCC, ccRCC;
